# Custom-made semi-joint prosthesis replacement combined ligament advanced reinforcement system (LARS) ligament reconstruction for the limb salvage surgery of malignant tumors in the distal femur in skeletal immature children

**DOI:** 10.3389/fped.2023.1168637

**Published:** 2023-06-21

**Authors:** Pengfei Zan, Jiakang Shen, Kaiyuan Liu, Hongsheng Wang, Zhengdong Cai, Xiaojun Ma, Wei Sun

**Affiliations:** Department of Bone Tumor Surgery, Shanghai General Hospital, Shanghai Jiaotong University, School of Medicine, Shanghai, China

**Keywords:** semi-joint prosthesis, osteosarcoma, LARS ligament, limb salvage surgery, complications

## Abstract

**Purpose:**

To explore the application of Custom-made Semi-joint prosthesis replacement combined with Ligament Advanced Reinforcement System (LARS) ligament reconstruction for the limb salvage surgery (LSS) of malignant tumors in the distal femur and provide selections for the LSS of malignant tumors in skeletal immature children.

**Methods:**

A total of 8 children with malignant tumors in the distal femur who underwent Custom-made Semi-joint prosthesis replacement combined LARS ligament reconstruction for LSS from January, 2018 until December, 2019 in our bone and soft tissue tumor center were retrospectively recruited. The prosthesis related complications, oncological prognosis and knee function were observed, and the surgical efficacy was comprehensively evaluated.

**Results:**

The average follow-up time was 36.6 months (30–50 months). The average osteotomy length was 13.2 cm (8–20 cm) according to the preoperative imaging results and the length of the customized prosthesis. Two years after operation, the average MSTS-93 score was 24.4 (16–29) which indicated good limb functions. The range of motion of the knee was 0°–120°, with an maximum average of 100°. At last follow-up, the average height of the children increased by 8.4 cm (6–13 cm), and the average limb shortening was 2.7 cm (1.8–4.6 cm). One patient had wound complications in the early postoperative period, wound scab fell off to form superficial ulcer, in whom debridement and suturing were performed. One patient developed hematogenous disseminated prosthesis infection 2 years after surgery, and the prosthesis is now *in situ* with anti-infection treatment. One patient developed pulmonary metastasis during follow-up, and received chemotherapy and targeted therapy with lesion well controlled. At the last follow-up, there was no local tumor recurrence or prosthesis loosening.

**Conclusion:**

Under the premise of appropriate case selection, customized semi-joint prosthesis replacement combined with LARS ligament reconstruction provides a new option for LSS in children with distal femur malignant tumors. LARS ligament reconstruction ensures the stability and range of motion of the knee joint, which maximally preserves the epiphysis of the tibia side and the growth function of the tibia side, reduces the complications of limb length inequality in the long term and creates conditions for limb lengthening or total joint replacement in adults.

## 1. Background

Children are the most common population of primary malignant bone tumors, which often occur in the metaphysis of the long diaphysis, mostly in the distal femur. Tumor resection and reconstruction are challenging in the limb salvage surgery (LSS) for malignant bone tumors in children (1, 2). Recently, with the advances of imaging, neoadjuvant chemocherapy, surgical techniques and postoperative comprehensive treatment, the 5-year survival rate of patients with newly diagnosed osteosarcoma has exceeded 70%, in the meanwhile, limb function and quality of life have been greatly improved (3). At present, the commonly used limb salvage strategies include autologous bone transplantation, allogeneic bone transplantation, allogeneic prosthesis composite construction, tumor bone inactivation grafting, bone transport with external fixation, and metal prosthesis reconstruction (4, 5). Among them, metal prosthesis reconstruction is the most widely used strategy in the clinical practice, which is easily operated and can provide immediate mechanical stability to achieve early functional rehabilitation after surgery (6). However, how to preserve of the growth function of the affected limb to the fullest extent and reduce the extent of the limb length discrepancy (LLD) is the focus and difficulties of current research (7).

Previous studies suggested that limb salvage surgery offered significantly better functional outcomes and quality of life compared to amputation, even in patients with distant metastasis (8). So, limb salvage has become a major surgical option in many cases and the development of reconstructive methods. While treating skeletally immature pediatric patients with such reconstructive methods is enormously challenging, especially in patients younger than 10 years old (9). The optimal way to complete tumor resection and reconstruction is still under discussion. It is often necessary to remove the entire distal femur to achieve a complete resection boundary when the distal femur tumor invades the metaphysis and crosses the epiphyseal line. The reconstruction strategy should take into account the function of the knee joint as well as the growth function of the tibia side. In the early stage, some surgeons proposed semi-joint prosthsesis replacement to reconstruct structural defects after resection of the malignant bone tumors around the knee joint in skeletal immature children and achieve impressive clinical outcomes (10). Whereas, due to the destruction of the ligament system of the knee joint, the stability of the knee joint in the early period is poor, thus external plaster fixation or brace protection is needed to restrict the joint activity, which delays the rehabilitation of the knee joint, long-term range of motion of the knee joint is lost in a large extent. In this study, we introduced the concept of ligaments reconstruction strategy of sports medicine, combined customized semi-knee prosthsesis replacement with Ligament Advanced Reinforcement System (LARS) surrounding to reconstruct the bone defects and cruciate and collateral ligaments, better clinical outcomes were anticipated.

From January 2018 to December 2019, 8 children diagnosed with primary malignant tumors in the distal femur thus received customized semi-knee prosthesis replacement combined with LARS ligament reconstruction were enrolled in our Bone and Soft Tissue Cancer Center. Objectives were: (1) to investigate the advantages and disadvantages of semi-knee prosthsesis replacement in the LSS for the distal femur malignant tumors in skeletal immature children; (2) to evaluate the functional outcomes and complications of the knee joint after semi-knee prosthesis replacement with LARS ligament reconstruction, and compare them with other reconstruction methods.

## 2. Materials and methods

### 2.1. Inclusion and exclusion criteria

#### 2.1.1. Inclusion criteria

(1) the children with a malignant tumor of the distal femur, aged between 6 and 12 years; (2) preoperative x-ray, CT, and MRI images confirmed that the malignant tumor of the distal femur invaded the posterior segment of the stem and crossed the epiphysis; (3) no tumor progression and no occurrence of lung metastases during preoperative neoadjuvant chemotherapy; (4) distinct tumor border and complete resection could be obtained; (5) a minimum of 2 years of postoperative follow-up.

#### 2.1.2. Exclusion criteria

(1) the tumor soft tissue mass invaded important blood vessels and nerves; (2) the early occurrence of lung metastasis from the tumor.

### 2.2. General data

Eight children with a malignant bone tumor of the distal femur, who were admitted successively to our Bone and Soft Tissue Tumor Center between January 2018 and December 2019, were included in the present study. After preoperative imaging evaluation, reconstruction was performed using a customized hemi-knee prosthesis replacement with a composite LARS ligament. The included patients comprised five boys and three girls, with a mean age of 9.6 years (age range: 6–12 years). Preoperative radiographs, 3D CT of the distal femur, MRI of the femur, CT of the chest, and bone scans were available for all included patients. Among these patients, 8 children had preoperative biopsies suggestive of osteosarcoma and were routinely treated with 4 rounds of preoperative neoadjuvant chemotherapy (methotrexate, doxorubicin, and cisplatin), customized hemi-knee prosthesis with LARS ligament reconstruction, and 8 rounds of postoperative adjuvant chemotherapy. The present study was approved by the ethics committee of our hospital, and the informed consent form for participation in the study was signed by the parents of all the included patients.

### 2.3. Surgical approach

**Figure 1:**
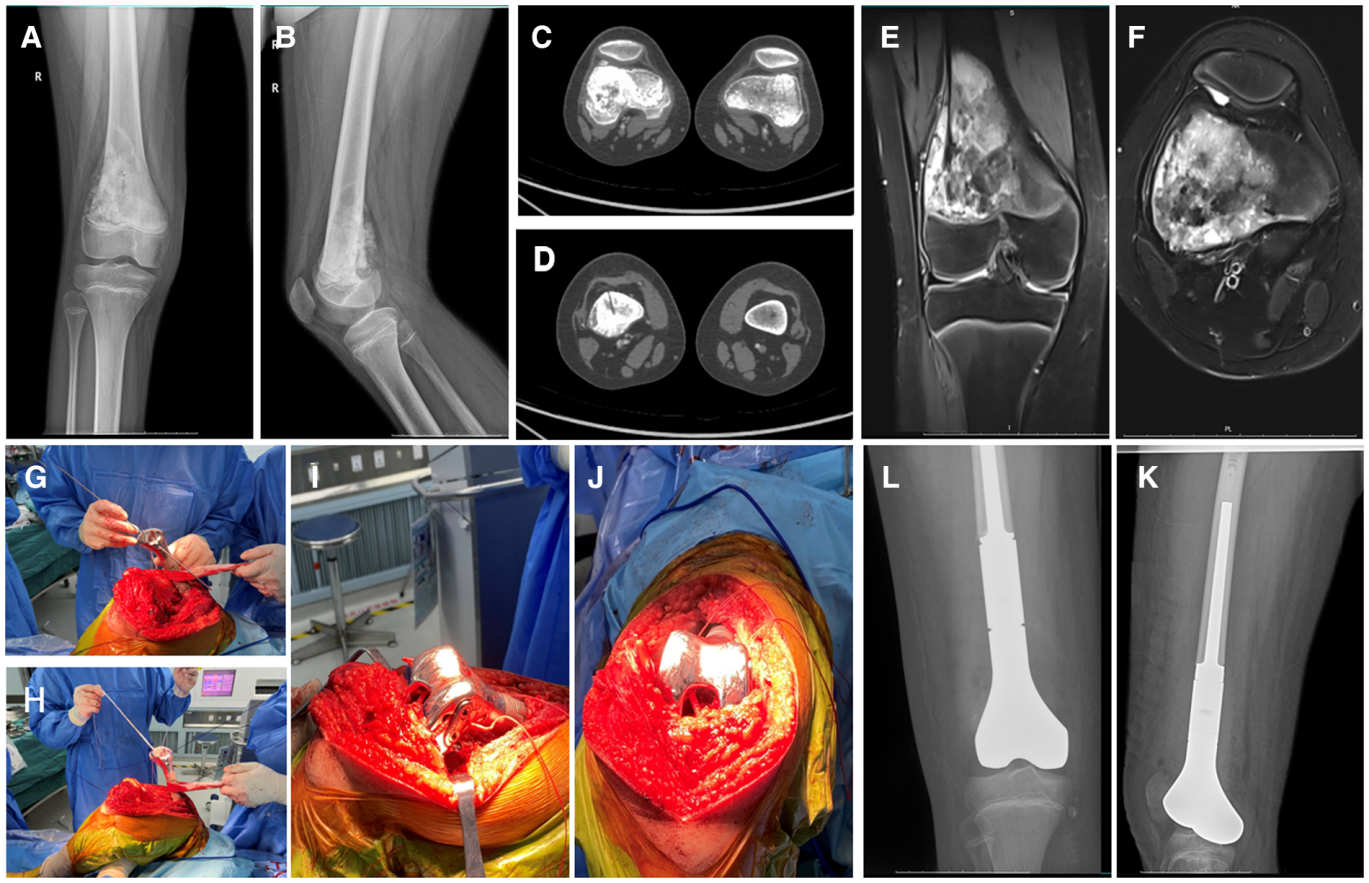
12 years old, male, osteosarcoma of the right distal femur, semi-joint prosthesis replacement combined ligament advanced reinforcement system (LARS) ligament reconstruction. (**A,B**) The radiograph of an osteosarcoma involving the metaphyseal region of the distal femur; (**C,D**) Preoperative CT indicated Osteoblastic destruction of the distal femur; (**E,F**) Preoperative MRI indicatedan abnormal signal area at the distal end of the right femur with a lateral soft tissue mass and tumor invasion of the epiphyseal line; (**G,H**) Methods of intraoperative LARS ligament reconstruction; (**I,J**) Appearance after LARS ligament reconstruction; (**K,L**) Postoperative x-ray of femur indicated that the prosthesis was in good position.

**Figure 2:**
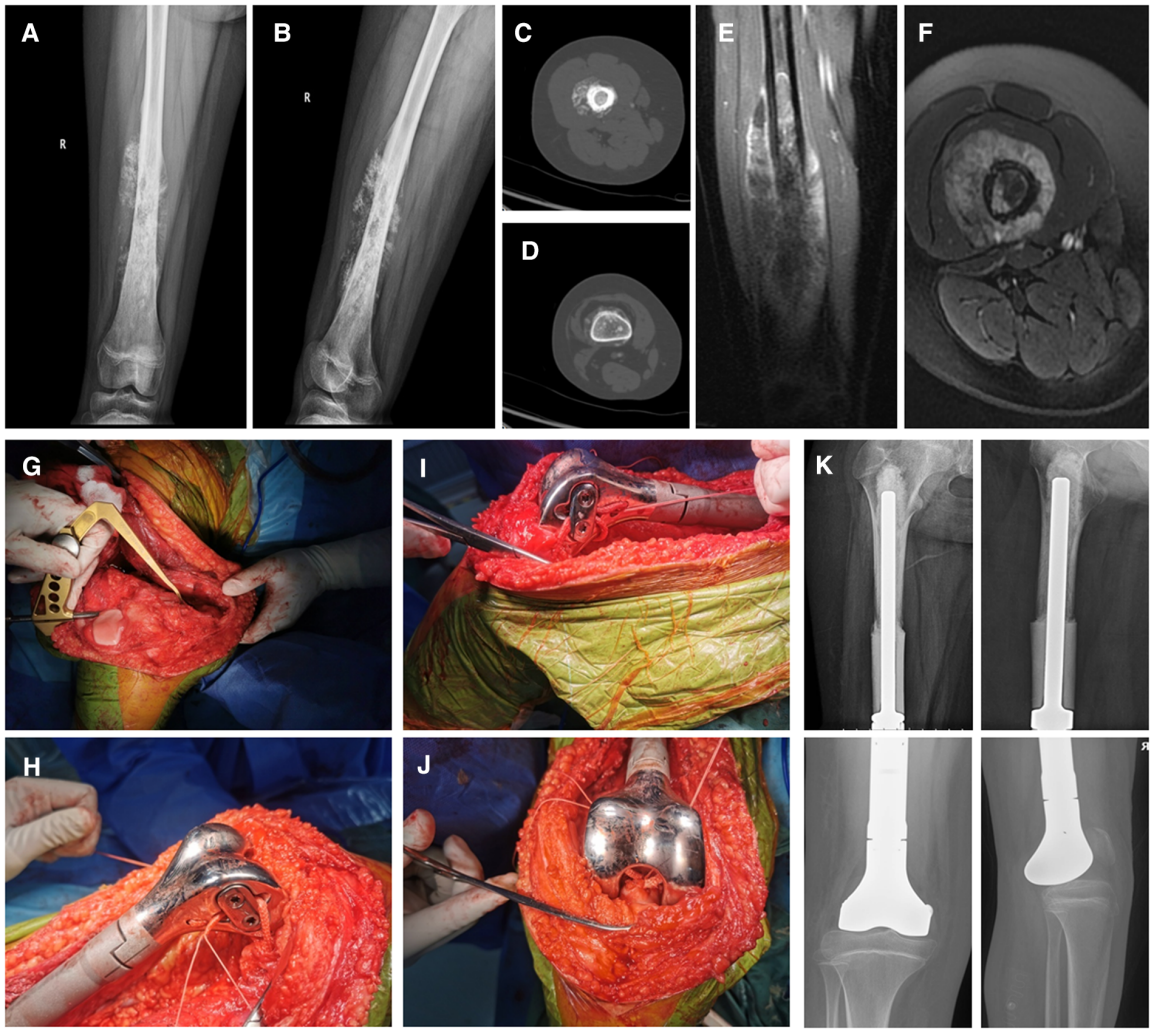
12 years old, female, osteosarcoma of the right distal femur, semi-joint prosthesis replacement combined ligament advanced reinforcement system (LARS) ligament reconstruction. (**A,B**) Preoperative radiographs of the femur revealed space occupation, bone destruction, and periosteal reaction of the right distal femur; (**C,D**) Preoperative CT revealed osteoblastic destruction of the right distal femur; (**E,F**) Preoperative MRI revealed an abnormal signal area in the right distal femur with a peripheral soft tissue mass and tumor invasion of the epiphyseal line; (**G–J**) The method of intraoperative LARS ligament reconstruction and its appearance after reconstruction; (**K**) Postoperative x-ray of femur indicated that the prosthesis was in good position.

1. Preoperative preparation: After the confirmation of the diagnosis of all included patients based on imaging and pathology, preoperative neoadjuvant chemotherapy was routinely performed 4 times, and imaging was performed again prior to the 4th chemotherapy session to assess tumor progression. The copies of the 3D CT and MRI images were sent to the company for customization of the semi-joint prosthesis, which was discussed by both the engineer and the surgeon.
2. Surgical operation: (1) Anesthesia and surgical approach: After the successful administration of general anesthesia, the patient was placed in the supine position. The puncture point was marked preoperatively, and an anterior approach to the knee was routinely adopted, distal to the tibial tuberosity and proximal to the mid-thigh. (2) Tumor resection: The perforator channel was removed in a shuttled shape. The medial and the lateral collateral ligaments and the anterior and posterior cruciate ligaments were severed at the articular surface of the distal femur, while carefully protecting the medial and lateral menisci, according to the principles of tumor resection. The blood vessels and the nerves were dissected and separated at the posterior aspect of the knee joint. The femur was incised at 2 cm from the tumor at the mid-femur and measured at the preoperative planned osteotomy length, following which the tumor was removed intact. (3) Installation of the prosthesis: The length of the limb was measured, and the affected limb was lengthened appropriately to ensure soft tissue coverage. The proximal femur was expanded, following which a trial mold of the prosthesis was fitted. The bone cement was prepared, the prosthesis was fixed to the proximal femur, and the internal and external rotation of the prosthesis according to the trajectory of the knee joint was determined. (4) LARS ligament reconstruction: The medial and lateral collateral ligaments and the anterior and posterior cruciate ligaments of the knee were located anatomically. A cruciate ligament reconstruction channel was created using sports medicine, while separate anterior and posterior cruciate ligament tunnels were created at the proximal tibia, and an entire LARS ligament was tied around the hemi-prosthesis and the proximal tibia. The anterior and posterior cruciate ligaments and the medial and lateral collateral ligaments were reconstructed. The ends of the ligaments were fixed in pre-determined grooves on the sides of the hemi-knee prosthesis using a small plate and two screws. The knee was mobilized intraoperatively to ensure appropriate knee extension and flexion. The knee lateral stress test was performed to confirm the stability of the knee joint right after the ligament reconstruction. The intraoperative images of the LARS ligament reconstruction are depicted in Figures 1G–J and Figures 2G–J. (5) The surgical incision was rinsed and sutured, which marked the completion of the operation. A typical case is depicted in Figures 1, 2.

### 2.4. Postoperative management

After surgery, it is recommended to remove the drainage tube when it drains less than 100 ml within 24 h, which usually occurs within 3 days after the surgery, to prevent retrograde infection. The patients were administered cefuroxime (cefaloxime) postoperatively as the anti-inflammatory agent until 72 h. After the drainage was removed, the active and passive joint movements were commenced with the assistance of a rehabilitation therapist. The patient was able to walk partially with the aid of a walker from 4 weeks after the surgery, and this was allowed until the patient could walk normally with full weight bearing. The knee was at a degree of 30 of constrain initial with brace and patients were allowed to start active and moderate passive physical therapy two weeks postoperatively. Postoperative chemotherapy was administered 2 weeks after wound healing. The chemotherapy regimen was formulated based on the results of the postoperative pathological tumor necrosis rate test, and 8 rounds of conventional postoperative chemotherapy were performed.

### 2.5. Follow-up and the indicators of efficacy evaluation

After surgery, the patients were routinely treated with adjuvant chemotherapy, and regular post-surgery follow-up reviews were conducted at 1 month, 2 months, 3 months, and every 3 months thereafter. Wound and prosthetic complications were assessed through physical examination, x-ray or CT scans, ultrasound examination, etc. The occurrence of lung metastases was ruled out based on a CT lung examination. The local and systemic tumor control was routinely assessed by performing bone scans 6 months after the surgery. Knee mobility was evaluated based on ROM, performed at two years postoperatively, and the MSTS-93 score was used to evaluate the function of the affected limb (11). The scale features pain, limb function, satisfaction, and imaging of the lower limb parameters (brace assistance, walking, and gait), and a total score of 30 may be obtained. A score of 80%–100% of the total score is considered excellent, a score of 60%–79% of the total score is considered good, and score of 40%–59% of the total score is considered fair, while a score <40% of the total score is considered poor.

## 3. Results

### 3.1. General information

All eight children with malignant tumors of the distal femur, who were included in the present study, were treated successfully using tumor resection and customized hemi-knee prosthesis replacement combined with LARS ligament reconstruction. All eight treated children also received thorough follow-ups. The average follow-up duration was 36.6 months (30–50 months). The average operation duration was 3.5 h (3–5 h). The average intraoperative blood loss was 400 ml (200–800 ml).

### 3.2. Tumor regression

The extent and the boundary of the tumor resection were determined based on the preoperative x-ray, CT, and MRI images. The bone was osteotomized 2 cm from the proximal lesion of the tumor during the operation. All pathological margins were negative postoperatively. In the postoperative follow-up period, none of the children presented local recurrence or distant metastasis, except for one case that developed lung metastasis.

### 3.3. Clinical function, limb length, and imaging evaluation

In the follow-up at two years postoperatively, the range of motion (ROM) of the knee joint was measured, and the MSTS-93 score was used to evaluate the postoperative function of the affected limb. The ROM revealed a maximum knee flexion of 120° and a minimum knee flexion of 80°. All children, except one, presented 5° of extension restriction, and all could be straightened. The average range of motion was 0°-100°. The MSTS-93 score achieved for the best function was 29, while that for a poor function was 16. There were 6 cases of “excellent” function, 1 case of “good” function, 1 case of “acceptable” function, and 0 cases of “poor” function. The mean MSTS-93 score was 24.4 (range: 16–29). In the last follow-up, the child's height had increased by 6–13 cm, with a mean increase of 8.4 cm, and the affected limb had shortened by 1.8–4.6 cm, with a mean decrease of 2.7 cm (Table 1), compared to the opposite side. The imaging results confirmed that the child's prosthesis was well positioned, and no loosening of the prosthesis had occurred.

**Table 1:** Baseline information and follow—up results.

	Gender	Age	L or R	Follow-up	Complication	ROM (°)	MSTS-93	LLD (cm)	Growth of proximal tibial epiphyseal plate
1	Male	12	R	50	Superficial ulcer treated with early wound dressing and debridement	0–100	24	2.2	Normal
2	Male	8	L	43	None	0–90	23	1.9	Normal
3	Female	12	R	40	None	0–120	28	4.6	Normal
4	Male	11	R	39	Pulmonary metastasis, undergoing chemotherapy	5–100	24	2.5	Normal
5	Female	8	L	37	None	0–105	27	2.1	Normal
6	Male	6	R	36	None	0–100	24	2.6	Normal
7	Female	11	R	34	Periprosthetic infection treated with active anti-infective treatment	0–80	16	3.9	Normal
8	Male	9	L	30	None	0–105	29	1.8	Normal

### 3.4. Complications

One child presented early postoperative wound complications, with the formation of a superficial ulcer after crusting. This case of complication was treated with early wound dressing and debridement, followed by suturing at the end of chemotherapy. In another case, the child developed a cold and fever along with inflammatory pulmonary manifestations two years after the surgery, which was followed by erythema and pain in the knee joint of the affected limb. The diagnosis of periprosthetic infection was confirmed bacteriologically, and the prosthesis was maintained in place while administering active anti-infective treatment. One of the children presented with metastatic lung lesions at the follow-up conducted one and a half years after the surgery. This case is currently receiving chemotherapy and targeted therapy, and good control of the lesions is being achieved. No local tumor recurrence was observed at the last follow-up in any of the children, and no cases of prosthesis loosening were recorded.

## 4. Discussion

Limb-sparing surgical prosthesis replacement for malignant tumors is a common surgical procedure in adults. However, in rapidly-growing children and adolescents, post-tumor resection and reconstruction remain challenging and debatable in terms of accommodating a balance of limb length and durable limb function (12). Currently, several forms of limb-preserving reconstruction are available for children, including prosthetic replacement, biological reconstruction, arthro-fusion, rotational molding, or amputation. In these methods, the growth of the lower limb is largely dependent on the periprosthetic region, with the distal femur growing approximately 9 mm per year, or 70% of the entire femur, while the proximal tibia growing approximately 6 mm per year, or 60% of the entire tibia (7). Therefore, the desired limb length shortening and the options for achieving limb isometric length are critical factors when selecting the method of reconstruction.

Children with malignant tumors experience a wide range of postoperative issues after limb-sparing surgery due to their rapid growth. In particular, complications such as unequal limb length or distant prosthetic loosening and infection could lead to severe physical disability (6, 13). Limb dissection surgery (including rotational molding or amputation) is commonly used in patients with Enneking stage III, local recurrence, or poor response to chemotherapy. This method is also used for younger children (<9 years of age). However, limb disarticulation surgery presents cosmetic, emotional, and functional problems, which several of the affected children and their parents find difficult to overcome (14). Metal prosthesis replacement provides good stability in the immediate postoperative period and assists the child in regaining early rehabilitation and function. However, in the long-term scenario, the prosthesis could become loose, fractured, or infected, requiring an undesired second-stage revision surgery (6, 15). The team previously summarized and analyzed 20 cases of revision after oncological knee prosthesis replacement, among which prosthesis loosening occurred in 25% of the cases, structural failure of the prosthesis occurred in 55% of cases, and periprosthetic infection occurred in 15% of cases. These observations suggested that structural failure of the prosthesis and prosthesis loosening were the main causes of distant revision surgery (16). While an invasive lengthenable prosthesis has been approved for children with a growth spurt, its 5-year prosthetic survival rate is just 59.4%. In addition, the procedure is dynamic and requires an incision under general anesthesia for each lengthening, and at certain times, even the removal of a more bony or fibrous tissue to achieve the desired lengthening, which significantly increases the risk of infection (17). Aseptic loosening is another major complication that occurs in this kind of prosthesis. Zou's team reported 45 cases of children whose height was lengthened with a non-invasive extendable prosthesis, and among these cases, seven were reported to have developed aseptic loosening of the prosthesis (18). Recently, novel non-invasive lengthenable prostheses have received widespread attention both nationally and internationally. These prostheses may be lengthened using an extracorporeal magnetic field, thereby preventing the risk of complications from surgical lengthening. However, the cost of this kind of prosthesis is high while its lifespan is not long due to the overemphasis on lengthening efficacy (19). So far, the application of this prosthesis in limb preservation surgery for malignant bone tumors in children has remained in the exploratory stage only.

In children with malignant tumors of the distal femur not crossing the epiphysis, the epiphysis may be preserved after a precise resection of the tumor segment, and the reconstruction may be conducted in a variety of ways. Previous studie have achieved excellent results with the customized replacement of the epiphysis-preserving prosthesis through the navigated resection of the tumor segment (20). However, in cases where the tumor crosses the epiphysis, the entire distal femur has to be removed to ensure a secure tumor border, and this may lead to numerous problems for the reconstruction. In 2005, Professor Yang's team (10) designed a hemi-knee prosthesis that did not destroy the contralateral normal epiphysis. This prosthesis could be appropriately lengthened in design to extend the affected limb as much as possible during the stage I surgery to reduce the degree of limb inequality in adulthood. Three patients in the study group were followed up for over 3 years, and a height gain of 6–11 cm and a shortening of 2.6–4.8 cm of the affected limb was achieved, compared to an average shortening of 9 cm after total joint replacement in children. In another study using non-hinged endoprosthesis, the actual LLD at the femur was 5.3 cm, which shows that the semi-joint did not affect too much on the LLD when compared to other reconstruction options (21). While this prosthesis was designed with holes for the reconstruction of the patellar ligament and the medial and lateral collateral ligaments, the reconstruction of the ligament after severance with the metal prosthesis tended to lack stability. In addition, this group of patients did not undergo reconstruction of the anterior and posterior cruciate ligaments, resulting in poor joint stability in the early postoperative period, which necessitated plaster or brace immobilization to restrict joint movement. This led to muscle atrophy and poor long-term joint functional rehabilitation. In the present study, the design of the hemiarthroplasty was refined by adding an anterior and posterior cruciate ligament reconstruction channel to the femoral prosthesis and incorporating a small plate and screws for medial and lateral collateral ligament fixation. The reconstruction of the anterior and posterior cruciate ligaments and the medial and lateral collateral ligaments was performed using LARS ligament wraps, and the knee joint was moved intraoperatively to ensure reliable knee flexion and extension. In order to achieve the anterior and posterior cruciate ligament fixation, a tunnel had to be created on the lateral tibial side for fixation. Although the lateral tibial epiphysis was partially destroyed, the diameter of the tunnel was just 3 mm, which would theoretically not impair the epiphyseal growth function. The postoperative shortening of the affected limb in this group was observed to be comparable to that achieved in the study reported by Prof. Yang's group. An average shortening of 2.7 cm was achieved, the range of motion of the knee joint was increased greatly, and the MSTS-93 score was also satisfactory. In addition, considering less influence on LLD when compared to other reconstruction options, the semi-joint did not affect too much on the the appearance of lower extremities.

Previous literatures showed that compared to other reconstruction methods, semi-constrained knee prostheses have a similiar impact on ROM (12). However, it is important to note that different studies may have differing results, and the specific circumstances of each patient may also affect the surgical outcomes. Therefore, when formulating a surgical plan, doctors need to conduct a comprehensive evaluation and discussion to determine the most suitable treatment plan for the patient.

The prosthesis used in the present study, however, has one disadvantage. The hemi-knee prosthesis is theoretically temporary, and when the child's skeleton matures, it may require lengthening or replacement with a total joint prosthesis. Nonetheless, this prosthesis is a promising option for the reconstruction of malignant knee tumors in rapidly growing children, as it allows for early functional rehabilitation of the knee and maximization of the maintenance of limb length, thereby facilitating future limb lengthening and total joint replacement.

## 5. Conclusion

Knee malignancies in children present great challenges during reconstructions, and treatment decisions are, therefore, a joint struggle for the doctors and the affected families. In children with distal femoral malignant tumors invading the epiphysis, hemi-knee prosthetic replacement with LARS ligament reconstruction is an excellent option for preserving the contralateral normal epiphysis, maximizing the limb length, and reducing the degree of limb inequality in adulthood. Good knee stability at an early stage facilitates rehabilitation exercises, improves limb function, and provides better conditions for subsequent limb lengthening or total joint replacement.

## Data Availability

The original contributions presented in the study are included in the article, further inquiries can be directed to the corresponding author.
